# A technology-assisted health coaching intervention vs. enhanced usual care for Primary Care-Based Obesity Treatment: a randomized controlled trial

**DOI:** 10.1186/s40608-018-0226-0

**Published:** 2019-02-04

**Authors:** Clare Viglione, Dylaney Bouwman, Nadera Rahman, Yixin Fang, Jeannette M. Beasley, Scott Sherman, Xavier Pi-Sunyer, Judith Wylie-Rosett, Craig Tenner, Melanie Jay

**Affiliations:** 1 Veteran Affairs New York Harbor Healthcare System & NYU School of Medicine, New York, USA; 2 NYU School of Medicine & Veteran Affairs New York Harbor Healthcare System, New York, USA; 3 NYU Langone Health & Veteran Affairs New York Harbor Healthcare System, New York, USA; 4 New Jersey Institute of Technology, New York, USA; 5 NYU School of Medicine, New York, USA; 6 Columbia University Medical Center, New York, USA; 7 Albert Einstein College of Medicine, New York, USA

**Keywords:** Obesity, Primary care, Behavior change, Feasibility, Weight loss, Lifestyle, Health coach, Telehealth, Diet, Physical activity

## Abstract

**Background:**

Goals for Eating and Moving (GEM) is a technology-assisted health coaching intervention to improve weight management in primary care at the Veterans Health Administration (VHA) that we designed through prior rigorous formative studies. GEM is integrated within the patient-centered medical home and utilizes student health coach volunteers to counsel patients and encourage participation in VHA’s intensive weight management program, MOVE!. The primary aim of this study was to determine the feasibility and acceptability of GEM when compared to Enhanced Usual Care (EUC). Our secondary aim was to test the impact of GEM on weight, diet and physical activity when compared to EUC.

**Methods:**

Veterans with a Body Mass Index ≥30 kg/m^2^ or 25–29.9 kg/m^2^ with comorbidities (*n* = 45) were recruited in two phases and randomized to GEM (*n* = 22) or EUC (*n* = 23). We collected process measures (e.g. number of coaching calls completed, number and types of lifestyle goals, counseling documentation) and qualitative feedback on quality of counseling and acceptability of call duration. We also measured weight and behavioral outcomes.

**Results:**

GEM participants reported receiving high quality counseling from health coaches and that call duration and frequency were acceptable. They received 5.9 (SD = 3.7) of 12 coaching calls on average, and number of coaching calls completed was associated with greater weight loss at 6-months in GEM participants (Spearman Coefficient = 0.71, *p* < 0.001). Four participants from GEM and two from EUC attended the MOVE! program. PCPs completed clinical reminders in 12% of PCP visits with GEM participants. Trends show that GEM participants (*n* = 21) tended to lose more weight at 3-, 6-, and 12-months as compared to EUC, but this was not statistically significant. There were no significant differences in diet or physical activity.

**Conclusions:**

We found that a technology assisted health coaching intervention delivered within primary care using student health coaches was feasible and acceptable to Veteran patients. This pilot study helped elucidate challenges such as low provider engagement, difficulties with health coach continuity, and low patient attendance in MOVE! which we have addressed and plan to test in future studies.

**Trial registration:**

NCT03006328 Retrospectively registered on December 30, 2016.

**Electronic supplementary material:**

The online version of this article (10.1186/s40608-018-0226-0) contains supplementary material, which is available to authorized users.

## Background

The prevalence of obesity in the United States is high (39.5%) [1]. Modest weight loss through behavioral interventions can reduce disease burden and improve quality of life [2]. Clinical guidelines recommend that providers refer patients with obesity to multi-component lifestyle-based weight management programs (≥14 sessions over 6 months) [3, 4]. Veterans seen in primary care (PC) at the Veterans Health Administration (VHA) are systematically screened for obesity, and those with a body mass index (BMI) ≥ 25 kg/m^2^ are referred to the MOVE! Weight Management Program [5], an intensive, multi-component behavioral lifestyle intervention that adheres to clinical guidelines. However, fewer than 10% of eligible patients attend at least one session [6]. Thus, lifestyle-based weight management services at the VHA are underutilized and, similar to other settings; obesity is undertreated [7].

The average VHA patient visits their primary care provider (PCP) 3.6 times per year; thus PC serves as a critical intervention point to providing counseling and linkages between established programs [8]. However, PCPs have difficulty managing obesity due to negative attitudes [9], poor counseling competency [10], and lack of time [11]. Despite this, patients with obesity want PCPs to give them guidance about weight management and discussions with physicians about weight are associated with higher odds of weight loss [12, 13].

The United States Preventive Services Task Force (USPSTF) has recommended that providers use the 5As framework to counsel patients for weight management, and Medicare reimburses 5As counseling [4]. This framework promotes individualized counseling by guiding the provider to Assess risk and stage of change, Advise weight loss and behavior change, Agree on goals, Assist via addressing barriers (motivational interviewing), and Arrange to follow-up or refer patient for further treatment [14]. Through rigorous formative work, we designed the Goals for Eating and Moving Intervention (GEM) to deliver 5As counseling within the context of the Patient-Centered Medical Home model of primary care without overburdening PCPs and other members of the healthcare teams. Prior publications describe how we developed GEM iteratively using the Orbit model of behavioral intervention design [15] with formative methods including focus groups with Veterans [16], key informant interviews with staff [9], software usability testing [17] and pilot-testing [17].

The primary aim of this pilot randomized controlled trial (RCT) was to explore feasibility and acceptability of the GEM intervention. The secondary aim was to test preliminary outcomes of GEM on weight, diet and physical activity when compared to Enhanced Usual Care (EUC) at 3-, 6-, and 12-months.

## Methods

**Fig. 1:**
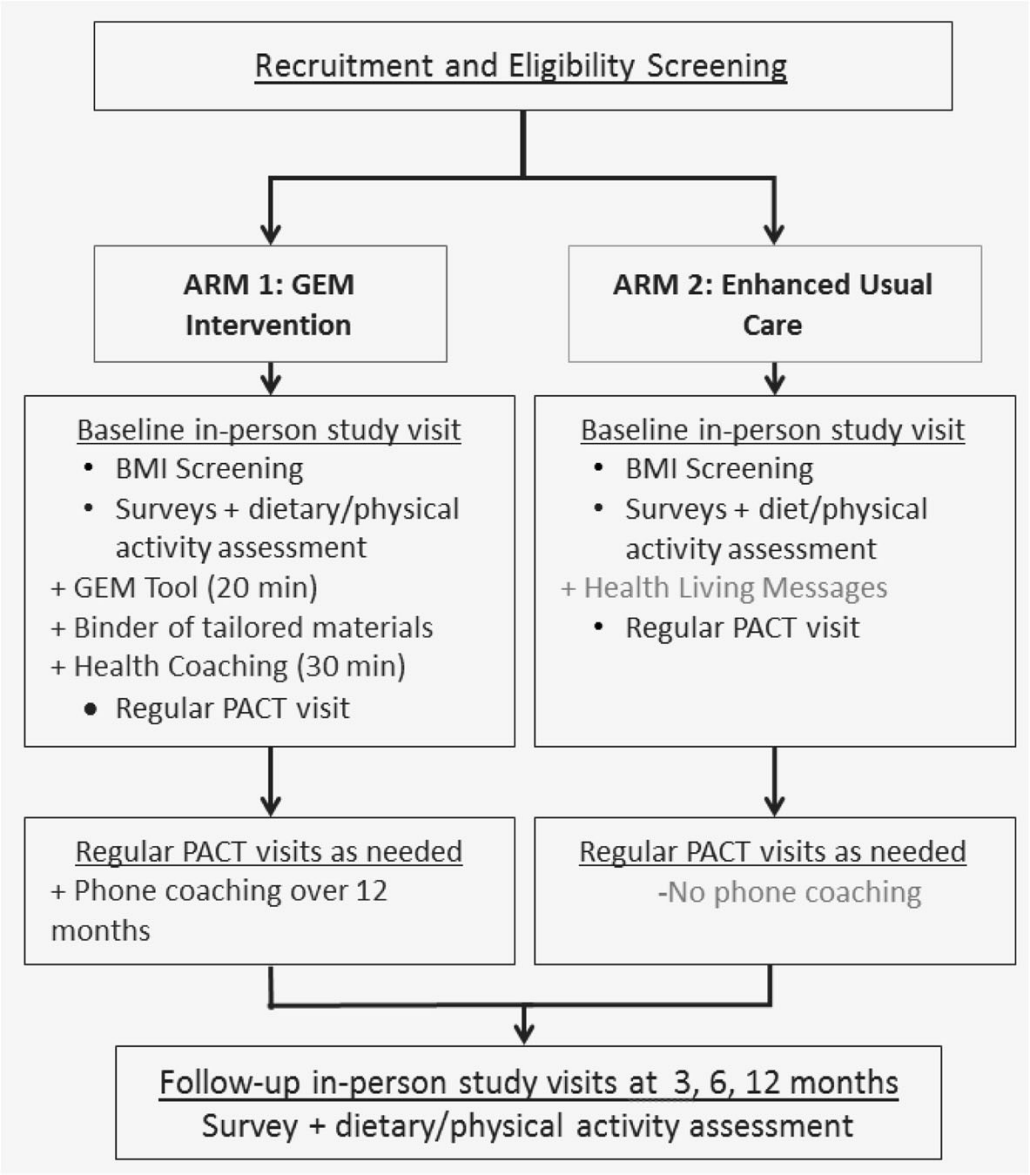
The Goals for Eating and Moving (GEM) Intervention vs. Enhanced Usual Care (EUC) study diagram

We conducted a pilot randomized controlled trial of the Goals for Eating and Moving (GEM) intervention compared to Enhanced Usual Care (EUC) (Fig. 1). This study was conducted in two phases in order to address issues with recruitment and health coach continuity identified during phase 1.

### Recruitment and participants

For both phases, we identified eligible patients from the Manhattan VA New York Harbor Healthcare System through a filtering process which involved 1) querying lists via the electronic medical record, Veterans Information System and Technology Architecture (VISTA), 2) confirming eligibility with their PCP, 3) mailing recruitment letters and 4) completing a telephone screening. Trained research staff completed the screening by reviewing the patient’s electronic medical record and completing a telephone eligibility questionnaire. Eligible patients were male and female Veterans between the ages of 18 and 69 with a body mass index (BMI) equal to or greater than 30 kg/m^2^ or with a BMI between 25 and 29.99 kg/m^2^ with at least one comorbidity. Comorbidities included hypertension, obstructive sleep apnea, high cholesterol, prediabetes, and metabolic syndrome. Additional eligibility criteria included having a prior PCP visit within the past year, regular access to a telephone, travel accommodations to the medical center, proficiency in English, and no weight loss greater than 5% in the past year. We also excluded patients who attended three or more MOVE! sessions within the past 12 months, were pregnant, had an illness affecting weight stability, had active psychosis or other cognitive issues, or a history of bariatric surgery. Inability to read at a fifth-grade level was an additional exclusion criterion.

During phase 1, participants were scheduled for a baseline visit on the same day preceding a routine PCP visit. Linking the study visit with the PCP visit severely limited the pool of eligible patients and created scheduling problems. Thus, during phase 2, we scheduled baseline visits independent of PCP visits. We obtained written consent from all participants in the study during their baseline visit. The study protocol and procedures were approved by the Veterans Health Administration New York Harbor Healthcare System Institutional Review Board. Follow-up data collection occurred at 3-months, 6-months and 12-months and participants were provided monetary compensation for time and travel to research study visits ($40 for baseline, $30 for 3- and 6-month visit and $60 for 12-month visit).

### Intervention

We used Software R (version 3.4.1) to conduct two-arm block-randomization of size 4, randomizing participants to GEM or EUC.

### GEM intervention

We developed the GEM intervention through rigorous formative research [9, 16]. The GEM intervention works within the VHA’s patient-centered medical home in which care is provided by Patient Aligned Care Teams (PACT). The main goal of GEM is to facilitate lifestyle-based weight management within PC by providing weight management counseling using the 5As framework [4] (Assess, Advise, Agree, Assist, Arrange) and increasing attendance to MOVE!. MOVE! is a national, intensive weight management program comprised of group-based support and skill-building lessons focused on self-monitoring, diet, physical activity, and weight loss for Veterans seen at the VHA. Veterans who attended MOVE! ≥3 times within an 8-week period demonstrated significant weight loss after 1–2 years [18]. Given time limitations on providers, we trained student health coaches to counsel patients and provide information on available VHA programs. Counseling was primarily conducted via telephone since telephone-based counseling can be as effective as in-person counseling for weight loss, and Veteran patients appreciate the convenience of telehealth modalities like e-mail messaging, telephone, and video conferencing to avoid lengthy scheduling calls and unnecessary travel to their local VA facility [19, 20].

GEM utilizes three components to deliver counseling: health coaches, the tablet-delivered GEM tool, and counseling from their PACT [17].

#### Health coaches

Health coaches are undergraduate and graduate pre-medical and public health students who volunteer 10–15 h per week as student coaches for 1–2 years. For this study, there were 11 student coaches (i.e. 4 undergraduate, 3 graduate, 3 post-baccalaureate and 1 pre-medical gap year student; 10/11 were female). They received 25 h of motivational interviewing and protocol training to build counseling skills in the context of GEM.

Health coaches worked within the VHA PACT and communicated directly with team members to facilitate care. They encouraged participants to join MOVE!. They met participants in person during the baseline visit and via telephone for follow up.

#### Baseline visits

The baseline visit with the health coach involved a one-hour session providing an overview of the tablet-delivered GEM tool (described below) and a counseling session. Health coaches taught the participant to use two self-monitoring tools—a pedometer and a food/physical activity diary. The health coach used Motivational Interviewing to explore motivation and barriers for losing weight, increasing physical activity, and making dietary changes. The health coaches documented their encounter in the electronic medical record with a note that generated a clinical reminder (described below) for the PCP. These notes were co-signed by the PCP and the PACT dietitian to facilitate communication.

#### Telephone coaching

Veterans received up to 12 coaching calls (~ 25 min each). Health coaches called participants to remind them to use food records and pedometers three days prior to each coaching session. Health coaches documented calls in the electronic medical record and these were co-signed by the PACT dietitian.

#### GEM tool

Veterans used the GEM tool at baseline to answer a series of questions and complete a goal-setting algorithm. The tool then generated tailored education materials that were printed and given to the participant in a binder as a personalized care plan. These materials facilitated counseling during baseline and telephone calls. The binder included SMART goal worksheets, standardized MOVE! handouts, information on health resources, and a GEM summary report. The summary report was entered into the participant’s electronic chart for their PCP to review.

#### PCPs

To assist PCPs with communication and documentation, we designed an electronic prompt within the VHA electronic medical record. This GEM-specific clinical reminder prompted the PCP to read the health coach’s notes, indicate whether they discussed the participants’ goals, and complete weight counseling documentation. This was designed to take 15 to 30 s to complete.

PCPs received a one-time training (15–20 min) prior to the start of study recruitment using an academic detailing approach. Training covered 1) GEM overview, 2) Supporting participant goals and addressing barriers (e.g. pain, depression), 3) Role of the health coach and 4) Demonstration of an electronic clinical reminder. The PCPs were asked to discuss goals and address barriers, communicate with health coaches, and document weight counseling.

### Enhanced usual care (EUC)

Participants randomized to EUC received a printed flyer about MOVE! and a “VA Healthy Living” brochure from a health coach. This pamphlet covered screening tests and immunizations, stress management, tobacco and alcohol use, and physical activity [21]. Handouts encouraged participants to write down and discuss goals with their PCPs.

### Measures

#### Feasibility and acceptability

We monitored intervention uptake and feasibility among GEM participants and their PACTs. We collected data on types of goals set at baseline (i.e. weight, nutrition, physical activity, and other), number and duration of calls, and number of times self-monitoring was reviewed during calls. Self-monitoring consisted of self-weighing, step count (via pedometer), and food record. Health coaches entered data about the coaching visits into REDCap. After the intervention was completed, research assistants (RAs) tallied the number of primary care clinical notes mentioning health behavior language (i.e. lifestyle, exercise, diet, physical activity, nutrition, obesity) and the number of GEM-specific clinical reminders that the PCPs completed. Coaching calls were audio recorded and fidelity was assessed using a standardized 25-item fidelity checklist (see Additional file 1) adapted from the ASPIRE trial [22]. Health coaches rated each other’s calls using checklists to monitor fidelity and provide ongoing feedback to ensure quality.

#### GEM participant feedback

To measure acceptability of GEM, participants rated the overall quality of counseling received on a 10-point Likert scale, evaluated the length of calls as too short, just right, or too long and provided open-ended feedback on quality of interactions with the health coach and impressions of the intervention, though data were not formally analyzed.

#### Weight and height

At baseline, 3-, 6-, and 12-month visits, the average of two weight measurements were taken using a Medline MDR500PHY Physician Digital Scale with Height Rod. Participants were instructed to take off shoes and heavy clothing prior to weighing. Height was measured at baseline.

#### Survey data collection

Survey data were collected in-person via the Research Electronic Data Capture system, REDCap [23], an online application designed for administering and managing survey data. Since the study was a pilot with limited staff, RAs were not blind to intervention arm.

#### Survey measures

The following survey measures were taken only at the in-person baseline visit:

- Health literacy: adapted from 3-item health literacy screener from Chow et al. [24].
- Food insecurity: using a 6-item Household Food Security Scale [25].

The following survey measures were taken during in-person study visits at baseline, 3- months, 6-months and 12-months:

- Intention to change: 3 items addressing intention to change (i.e. lose weight, eat healthier, and get more exercise) [26].
- Motivation to change: measured using 3 items, ‘How motivated are you to make changes to weight, diet and physical activity’ and responses were graded on a 10-point scale ((1) Not at all motivated to (10) Very motivated) [26].
- Self-Efficacy and Health Behavior: dietary self-efficacy was measured using an 8-item scale [27] and physical activity self-efficacy was measured using a 5-item scale [28].
- Diet: assessed with a validated 17-item screener [29] and the Behavioral Risk Factor Surveillance System Sugar-Sweetened Beverage module assessed frequency of sugary drink consumption [30].
- Physical activity: was measured using the 8-item Paffenbarger Physical Activity Questionnaire [31]. We extracted moderate and vigorous (MVPA) physical activity hours to calculate a weekly MVPA score.

### Statistical analysis

Wilcoxon rank sum tests assessed 3-, 6-, and 12-month changes in weight, diet, physical activity, and self-efficacy. Fisher’s exact test assessed achievement of 3.0% weight loss from baseline weight at the time of each study visit. Moreover, for mean weight change and percent weight change, multiple-imputations were conducted to account for missing data, assuming missing at random. In addition, in the GEM arm only, the relationships between number of health coaching calls received and baseline demographic covariates were examined using Spearman correlation coefficients for quantitative covariates and Kruskal-Wallis tests for categorical covariates, respectively.

## Results

### Demographics

**Fig. 2:**
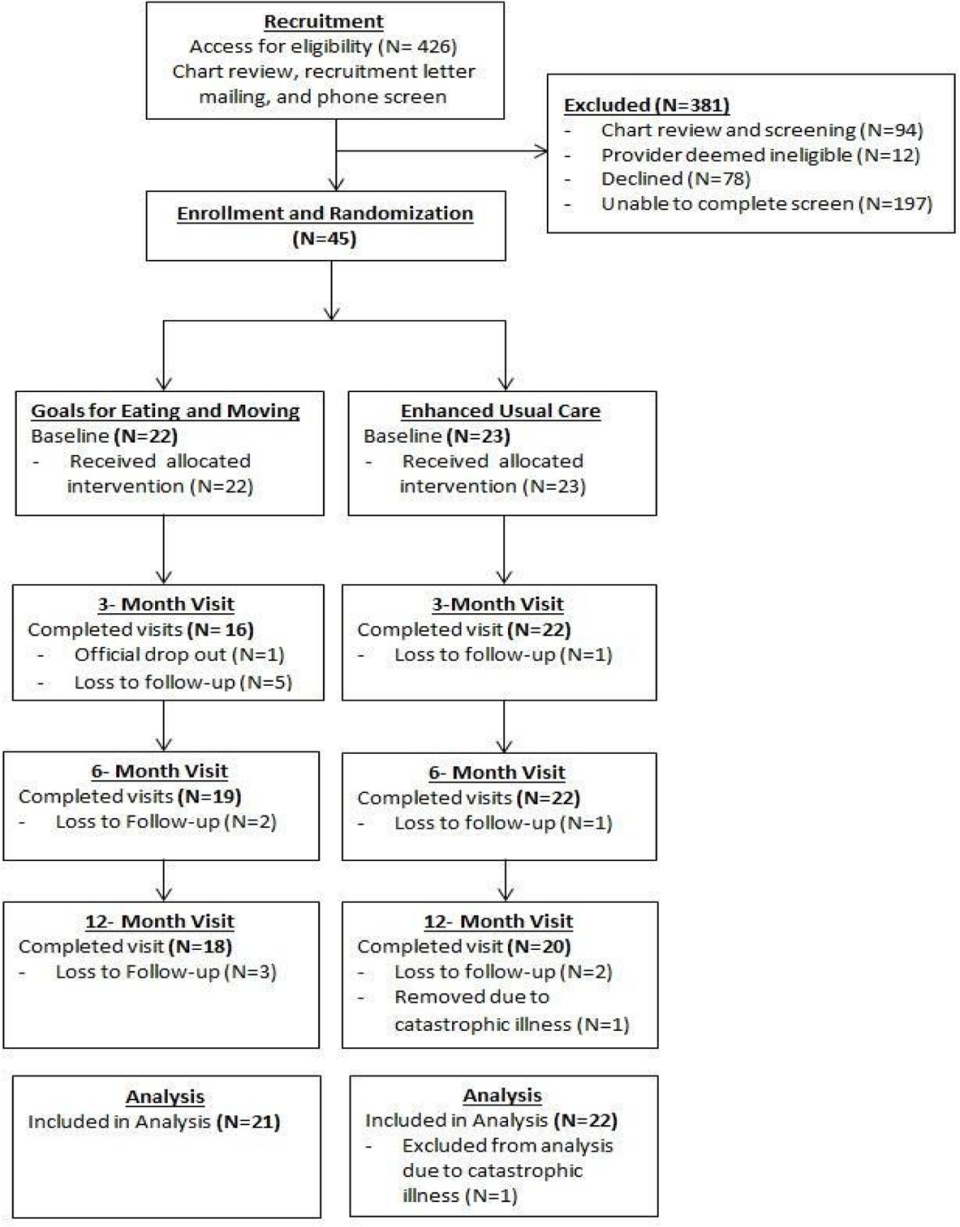
The Goals for Eating and Moving (GEM) pilot recruitment and randomization flowchart

Patients (*n* = 426) at the Manhattan campus of the VA New York Harbor Healthcare System were identified using the electronic medical record system (Fig. 1). Among them, 12 patients were deemed ineligible for the study by their PCP, and the remaining 414 were mailed recruitment letters and contacted by phone for screening. Of those, 139 completed screening procedures, 78 declined, and 197 were not reached. The 45 patients who met eligibility criteria and agreed to enroll in the study were randomly assigned to GEM (*n* = 22) or EUC (*n* = 23) and were scheduled for a baseline visit (Fig. 2). Of those recruited, there was one official drop-out from GEM and one participant was excluded from EUC due to catastrophic illness.

Demographic characteristics were similar in GEM and EUC. Mean age, BMI, gender, race/ethnicity, household income, medical conditions, military branch, intention to change, motivation, and self-efficacy were not significantly different between the two groups (Table 1).

**Table 1:** Participant Demographics

Characteristics		GEM (*n* = 21) Mean ± SD or n (%)	EUC (*n* = 22) Mean ± SD or n (%)
Age (years)		53 ± 10	56 ± 11
BMI (kg/m^2^)		31 ± 3	33 ± 6
Gender	Male	16 (76%)	13 (59%)
Race/Ethnicity^a^			
	Black or African American	11 (52%)	11 (50%)
	White or Caucasian	4 (19%)	4 (18%)
	Asian	2 (10%)	0 (0%)
	American Indian or Alaskan Native	1 (5%)	0 (0%)
	Hispanic	3 (14%)	6 (27%)
	Other	0 (0%)	1 (5%)
Annual Household Income			
	Less than $24,999	4 (19%)	8 (36%)
	$25,000 to $49,999	6 (29%)	7 (32%)
	$50,000 to $99,000	8 (38%)	6 (27%)
	$100,000 or more	3 (14%)	1 (5%)
Medical conditions^b^			
	Hyperlipidemia	29%	27%
	Hypertension	33%	46%
Branch of military	Army	57%	68%
	Navy	5%	14%
	Marines	14%	0%
	Air Force	24%	18%
Household food security^c^	Food secure	71%	73%
	Food insecure	24%	18%
	Hunger	5%	9%

^a^Categorical answer options for Race included Black, White, Asian, American Indian, and Other. Those who selected ‘Other’ and specified, ‘Hispanic’ were counted as Hispanic only, not as ‘Other’

^b^Medical data were collected by chart review and confirmed with participant self-report during baseline screening

^c^Food security was assessed using the 6-item validated scale, Household Food Security Scale (i.e. 2+ affirmative responses indicate food insecurity and 5+ affirmative responses indicate hunger) [25]

### Primary aim – Feasibility and acceptability

#### Goal-setting

Participants could set up to four lifestyle goals during the baseline visit and were prompted to discuss goals on subsequent telephone coaching calls. Weight goals were set by 95% (18/21) of the participants randomized to GEM, 95% (20/21) set a nutrition goal, 71% (15/21) set a physical activity goal, and 4.76% (1/21) set an “other” lifestyle goal. On average, GEM participants set 2.5 goals at the in-person baseline session and discussed 1.4 goals at each telephone coaching call session. There were no differences in number or type of goals set between phase 1 and phase 2 of GEM.

#### Telephone coaching calls

The average number of calls completed per participant throughout the intervention was 6.0 ± 3.7 (range: 0, 12) out of 12 attempted calls. On average participants reported their weight during 3.8 of completed calls (SD ± 3.1), reported their step count during 3.9 calls (SD ± 3.7) and reported using food diaries during 4.0 calls (SD ± 3.4). Of the 125 total completed phone calls, 119 included data on the call duration, which indicated the mean call duration was 23.6 min (SD ± 9.7 min). Of note, most participants in GEM had more than one coach throughout the intervention (mean = 1.8 coaches) due to turnover of coaches. Table 2 shows that men received more coaching calls than women in the study (6.81 ± 3.25 vs. 3.2 ± 3.96; *p* < 0.05). It also shows differences in number of calls received by race/ethnicity (*p* = 0.09) with Black/African American participants receiving the highest average number of calls. Other baseline demographic covariates, such as age, BMI, income and clinical variables, were not correlated with number of calls.

**Table 2:** Number of Coaching Calls Completed in the GEM Intervention Arm by Race/Ethnicity and Sex

	N	Mean Number of Calls	Standard Deviation	*p*-value
Race/Ethnicity				
American Indian	1	7.00	–	
Asian	2	3.50	2.12	
Black	11	7.73	3.04	
Hispanic	3	1.67	1.53	
White	4	5.25	4.65	
All	21	5.95	3.68	0.093*
Sex				
Female	5	3.20	3.96	
Male	16	6.81	3.25	
All	21	5.95	3.68	0.046*

*Kruskal-Wallis tests for categorical covariates

#### PCP visits

During phase 1, all 15 GEM participants visited their PCP on the same day as the baseline visit, whereas in phase two, GEM participants visited their PCP at any point during the 12-month intervention. The mean number of PCP visits per GEM participant and per EUC participant were the same (2.3 ± 1.2 (0, 5) vs. 2.3 ± 1.6 (0, 7)). Lifestyle counseling was documented in 68% of PCP visits per GEM participant and 66% of PCP visits per control participant. GEM-specific clinical reminders were completed in only 12% of PCP visits with GEM participants.

#### GEM participant feedback

GEM participants (*n* = 21) reported the quality of counseling received from coaches was very strong, or a 9.1/10 (using 10-point Likert scale) on average and 79% reported that coaching call duration was “just right.”

### Secondary aim: Weight and other outcomes

GEM participants (*n* = 21) tended to lose more weight at 3-, 6-, and 12-months as compared to EUC. The weight change comparisons between GEM and EUC were −0.8 ± 2.0 vs. 0.1 ± 2.4 (*p* = 0.07), −1.5 ± 3.1 vs. 0.2 ± 3.6 (*p* = 0.08) and −1.0 ± 4.2 vs. 0.7 ± 4.9 (*p* = 0.40) for 3-, 6-, and 12-months, respectively (Table 3). There were no statistically significant differences in dietary or physical activity changes between the two groups. The number of coaching calls completed was significantly associated with greater weight loss at 6-months (Spearman’s coefficient ρ = 0.71; *p* < 0.001). However, at 12-months this correlation was not significant (ρ = 0.29; *p* = 0.11). Additionally, during the 12-month intervention four participants in GEM and two participants in EUC reported attending MOVE! at least once.

**Table 3:** Changes in Weight and Self-Efficacy by Arm

	GEM (Phase 1 and Phase 2) *n* = 21	EUC *n* = 22	*p*-value
Weight			
3-month weight changes			
*3-month weight change (kg)* Mean ± SD^a^	−0.80 ± 1.95	0.07 ± 2.40	0.07
*3-month 3.0% weight loss*	6.25%	14.29%	0.62†
6-month weight changes			
*6-month weight change (kg)* Mean ± SD^a^	−1.52 ± 3.05	0.23 ± 3.64	0.08
*6-month 3.0% weight loss*	21.05%	23.81%	0.99†
12-month weight changes			
*12-month weight change (kg)* Mean ± SD^a^	−1.02 ± 4.16	0.74 ± 4.90	0.40
*12-month 3.0% weight loss*	27.78%	20.00%	0.71†
Self-efficacy			
3-month changes in self-efficacy			
*Dietary self-efficacy Mean ± SD*^*a*^	6.38 ± 8.92	3.81 ± 11.63	0.23
*Physical activity self-efficacyMean ± SD*^*a*^	3.06 ± 7.48	4.14 ± 11.39	0.74
6-month changes in self-efficacy			
*Dietary self-efficacy Mean ± SD*	5.16 ± 10.07	3.52 ± 9.76	0.17
*Physical Activity self-efficacy Mean ± SD*	3.84 ± 10.51	1.71 ± 11.76	0.32
12-month changes in self-efficacy			
*Dietary self-efficacy Mean ± SD*	4.89 ± 9.01	0.30 ± 13.69	0.22
*Physical Activity self-efficacy Mean ± SD*	−0.72 ± 7.04	1.80 ± 14.07	0.40

^a^*SD* Standard Deviation. Weight changes at 3-, 6- and 12-months are in comparison to baseline weight. Dietary self-efficacy was measured using an 8-item scale [27] with range (0–10) and physical activity self-efficacy was measured using a 5-item scale with range (0–10) [28]

† From Fisher’s exact test; ‡ from logistic regression; the others from Wilcoxon rank sum tests

## Discussion

In this pilot study, our first aim was to determine whether the GEM intervention is feasible and acceptable within the PC setting at the VHA. We found that trained student health coaches, in particular, are able to implement a brief health coaching protocol. Participants in the GEM pilot reported that, on average, quality of counseling received from student health coaches was high (average of 9.1 on a 10-point Likert scale) and call duration and frequency were acceptable. Feasibility was enhanced in phase 2 after we modified our protocol to allow baseline visits to occur separately from the patients’ PCP visit—this scheduling arrangement was more flexible and patient-centered making recruitment easier.

For our second aim, we explored weight and behavioral outcomes of GEM. We found that while we were underpowered to show significance, participants in GEM tended to have more weight loss than those in EUC. We also found that the number of calls was correlated with increased weight loss at 6 months. Although 1 kg of weight loss at 12- months is not considered clinically significant, 27% of GEM participants did have clinically significant amounts of weight loss at 12 months (i.e. 3% weight loss according to TOS guidelines 2013 [3]) as compared to 20% in EUC. This pilot study informed changes to the GEM intervention protocol (described below) that we anticipate will increase both intervention feasibility as well as the magnitude of weight loss.

This study builds upon prior work showing the potential effectiveness of delivering weight management interventions in primary care settings. The three Practice-based Opportunities for Weight Reduction (POWER) studies show that primary care-based weight management interventions can produce clinically significant weight loss [32–34]. Non-clinician staff (e.g., health coaches, medical assistants, health educators) were an important component of the three POWER interventions, and two incorporated technology. The GEM intervention is novel in that it is delivered within the patient-centered medical home model of care and utilizes pre-existing, onsite, weight management programs and resources. This pilot helped us to identify several challenges and led to improvements of the GEM intervention that we will test in future studies.

Challenge 1: Low provider engagement. The first challenge was low provider engagement. Only four of eight providers completed the GEM-specific clinical reminder and there was no significant difference in documentation of counseling between GEM and EUC. This indicates that participation in GEM may not have impacted provider-level counseling. While this intervention was designed with input from PCPs and healthcare teams, one short training intervention was probably not enough to affect practice change [17]. To address this, we have standardized contact that is more frequent with primary care providers; health coaches regularly attend PC team rounds and clinic meetings. More frequent contact may increase engagement in the intervention and physician-led counseling.

Challenge 2: Health coaching continuity. A second challenge was providing health coaching continuity with unpaid student volunteers. We expected that each health coach would be able to follow the same two or three participants throughout the intervention in order to enhance rapport between participant and health coach and facilitate continuity of conversation. Some volunteers had difficulty committing to a regular weekly schedule and the one-year requirement while others finished their commitment to the program in the middle of the study. Challenges with scheduling affected continuity of coaching, sometimes necessitating multiple health coaches per participant, while reducing the total number of calls conducted. During phase 2, we placed greater emphasis on the alignment of schedules between the assigned health coach and GEM participant and calls were monitored daily to ensure completion. These changes increased the number of calls completed in phase two and decreased the number of coaches needed per GEM participant. For future studies, we have instituted weekly health coach meetings with the health coaching team to review quality and fidelity of recorded counseling sessions as well as to discuss difficult cases. Given the high correlation between coaching calls and weight loss, health coaches now work in pairs to increase call completion with one coach designated as the primary coach and the other as the secondary to cover calls when the primary coach is unavailable. We also standardized and improved the health coach training which we anticipate will lead to improved weight loss outcomes. We added 15 additional hours of training (40 h total) to include more role-play and mock coaching sessions under the supervisor of a lead health coach. Health coaches are now required to pass a coaching exam before being assigned GEM participants.

Challenge 3: Low attendance to MOVE!. Clinical guidelines recommend that patients are referred to intensive, multicomponent behavioral interventions since these have the highest level of evidence for non-surgical interventions [3, 4]. Unfortunately, we expected more GEM participants would enroll in the MOVE! weight management program and this may have been a reason for lower than expected weight loss at 12-months. According to VHA data, participation in at least eight sessions of MOVE! within a 6-month period is associated with maintained weight loss at 3 years (− 2.2% of body weight) [18]. Locatelli et al. reviewed several established MOVE! programs and cited factors that may impact participant engagement including provider knowledge, campus reputation, inclusion of physical activity, engagement of physician champions, and multiple meeting times [35].

To address the low attendance rates, we have to develop a stronger, multifaceted approach to encourage participants to attend by supporting and reminding participants of MOVE! appointment times and advertising more broadly. We plan to develop stronger linkages with MOVE! and other programs including the MOVE! Telephone Lifestyle Coaching program [36], the Diabetes Prevention Program [37], and outside companies like Weight Watchers [38] which should provide more options for Veterans who encounter barriers related to work, scheduling and travel.

In addition to addressing challenges identified from this pilot study, we have translated the GEM tool and developed culturally appropriate materials for Spanish-speaking populations. Further research is needed to test efficacy and cost-effectiveness of the GEM intervention. As a next step, we will conduct efficacy studies at both the VHA and at non-VHA clinical sites. This study informed the development of a multi-site cluster-RCT of the GEM intervention (NIH # 1R01DK111928). We also will be testing an adaptation of the GEM intervention using Veteran peer health coaches (VA IIR HX-002119-01A2).

### Limitations

A major limitation of this study was that we had difficulty reaching potentially eligible patients via telephone or mail to screen for study entry and encountered high rates of patients declining to participate. Of 414 who were mailed letters and called, 197 (48%) were not reached despite multiple attempts and 78 (19%) actively declined participation. Thus, those that elected to participate in this research study may have been more motivated than the general population or more engaged in VHA health services. We were also underpowered to see statistically significant changes in weight loss and other clinically important outcomes. Other limitations arose from our reliance on self-reported data to assess behavior. Although we selected validated instruments, self-reported data may be susceptible to social desirability bias or recall bias [39, 40]. In this study, research assistants reported that participants had some difficulty quantifying their activity and reporting usual activity levels with the Paffenbarger Physical Activity Questionnaire [31].

## Conclusions

This pilot study demonstrated that a technology-assisted health coaching intervention for weight management was feasible and acceptable to patients within the patient-centered medical home model of care. If found to be effective, GEM could improve the delivery of weight management within primary care. By utilizing student health coaches integrated within the healthcare teams, this intervention could help alleviate some of the time and resource constraints found in primary care settings while increasing utilization of existing comprehensive weight management programs.

## Additional file

Additional file 1: Fidelity Checklist. Fidelity monitoring tool for GEM health coaches. (PDF 39 kb)

## Abbreviations

BMI: Body Mass Index
EUC: Enhanced Usual Care
GEM: Goals for Eating and Moving
MI: Motivational Interviewing
PACT: Patient Aligned Care Teams
PC: Primary Care
PCP: Primary Care Provider
POWER: Practice-based Opportunities for Weight Reduction
RA: Research assistant
RCT: Randomized controlled trial
VHA: Veterans Health Administration
VISTA: Veterans Information System and Technology Architecture
