# Supplementary material for: A technology-assisted health coaching intervention vs. enhanced usual care for Primary Care-Based Obesity Treatment: a randomized controlled trial
Source: BMC Obes. 2019 Feb 4;6:4. doi: 10.1186/s40608-018-0226-0 (PMC6360675; doi:10.1186/s40608-018-0226-0)
Supplement: Supplementary file 1 — Fidelity Checklist. Fidelity monitoring tool for GEM health coaches. (PDF 39 kb) [file 40608_2018_226_MOESM1_ESM.pdf]

HEALTH COACHING FIDELITY & SUPERVISION CHECKLIST

Date of Session: Participant ID: Rater's Initials:

Site: Session Number: Coach Initials:

DurationMin **1. How long was this session?** *(write in the number)* ____ minutes

Mode **2. Session Mode?**
__ a) Baseline __ b) Telephone

Met **3. Overall, do you feel that the objectives of the session were:** *(circle)*
*Automatic calculation from Summary Scores 1-3 in Section 5: (Range 0-30)

30 =*Fully Met* **25-29** = *Mostly Met* **20-24**= *Partly Met* **15-19** = *Somewhat Met* **<15** = *Not Met*

4. General Session characteristics

Fixed __Participant was fixed on one topic (would not move on)
OnTopic __Participant/group stayed on topic (minimal tangential conversation)
Short __Participant had to cut the session short/group ended early
Disruptive __Patient was disruptive
Abusive __Patient was abusive
PtInter __Interruptions (from patient's environment)
StInter __Interruptions (from staff member's environment)
Other __*Other:*____

5. Session Core Elements
KEY for Summary Scores 1-3 (should be no NAs for this section):
0: Did not cover = this topic or focus point did not happen at all
1: Partially covered = this happened to some extent, but not completely
2: Fully covered = the goal was met fully

Summary Score 1 (Baseline ONLY): Review – Baseline health coaching session

Binder __ a – Health Coach outlines content of the binder
WeightLossGoal __ b – Health Coach discusses weight loss goal
NutritionGoal __ c – Health Coach discusses nutrition goals (i.e. turns into SMART goal)
PAGoal __ d – Health Coach discusses physical activity goal (i.e. turns into SMART goal)
Barriers __ e – Health Coach discusses barriers to weight management
Handouts __ f – Health Coach refers to handouts to discuss weight management strategies and barriers
Resources __ g – Health Coach discusses intensive interventions (i.e. turns into SMART goal)
SelfMonitor __ h – Health Coach discusses self-monitoring tools (i.e. journaling, pedometer, smartphone apps)
SummaryB __ i – Health Coach summarizes session (i.e. reviews SMART goals, refer to PACT teams)
FollowupB __ j – Health Coach summarizes contact information and sets up follow-up appointments

Summary Score 1 (Telephone ONLY): Review – Goal attainment and self-monitor check-in

GoalAtt __ a – Health Coach prompts review of goal attainment (i.e., review of goals *versus* actual)
ResourcesUse __ b – Health Coach determines use of resources (and/or discusses barriers) and collects usage data
SelfMonitorUse __ c – Health Coach determines use of self-monitoring tools (and/or discusses barriers) and collects usage data
Succ/Fail __ d – Health Coach discusses successes/failures (facilitators/barriers) since last session
GoalUpdate __ e – Health Coach initiates problem-solving approach when necessary to address barriers and update goals
MTGnutrition __ f – Health Coach updates response to MTG nutrition questions
MTGphysical __ g – Health Coach updates response to MTG physical activity questions
Safety __ h– Health Coach elicits any study or non-study related medical or adverse events from the participant
SummaryT __ i – Health Coach summarizes session (i.e. reviews new SMART goals)
FollowupT __ j – Health Coach sets up follow-up appointments

Summary Score 2: Core Psycho-educational Content

MotivInterv __ a – Health Coach uses MI techniques to develop a self-management skills or change cognition

Tailored __ b – Health Coach tailors session by assessesing knowledge of topic, degree of relevance to their weight management practices, and increasing knowledge/skills

Summary Score 3: Action Planning and Session Wrap-up

Goal __ a – Health Coach prompts participant to identify SMART goals to work on before the next session

Confidence __ b – Health Coach aassesses participant’s motivation and self-confidence/readiness to follow-through on plan

Agreement __ d – Health Coach and participant come to agreement on small change goals

KEY for Summary Scores 7-10:

0: Did not demonstrate = this process objective or component was not demonstrated at all

1: Inconsistently Demonstrated = this happened to some extent, but not all of the time

2: Demonstrated consistently through entire session = objective was demonstrated consistently and appropriately throughout the entire session

9: NA

7. Summary Score 4: Interventionist Personal Characteristics

Didactic __ Health Coach delivered didactic material in a matter of fact and friendly way

Nonjudge __ Health Coach avoided judgmental feedback on participant(s) contributions

Empathy __ Health Coach responded empathically and accurately to individual or group member behavior (verbal, nonverbal)

8. Summary Score 5: Session Structure

Organized __ Health Coach came prepared and organized

Timing __ Time was allocated appropriately in order to cover the appropriate content focus points for that session

9. Summary Score 6: Session Flow

Process __ Health Coach addressed process (tangential) issues but did not allow them to disrupt content agenda

Modulation __ Health Coach modulated distractions (e.g., side bar conversations, interruptions by family members)

Delving __ Health Coach avoided delving too deeply into psychological issues

10. Summary Score 7: Engagement with Patients

Questions __ Health Coach facilitated discussion and interaction using open-ended questions, affirmations, reflections, summaries

Clarified __ Health Coach elicits clarification of participant(s)’ engagement by seeking feedback about didactic content

11. What activities or additional topics were added to any aspect of the session? *(Describe)*

12. Additional Comments
